# Breastfeeding support among re-hospitalized young children: a survey from Italy

**DOI:** 10.1186/s13052-023-01573-x

**Published:** 2024-01-08

**Authors:** Elena Scarpato, Guglielmo Salvatori, Michelangelo Barbaglia, Deborah Casero, Teresa Cazzato, Veronica Righetti, Annamaria Staiano, Riccardo Davanzo

**Affiliations:** 1 Department of Translational Medical Sciences, Section of Pediatrics, University Federico II of Naples, Naples, Italy; 2 Neonatal Intensive Care Unit, IRCCS Bambino Gesù Children’s Hospital, Rome, Italy; 3 Pediatric Unit, “Castelli” Hospital, ASL VCO, Verbania, Italy; 4 Pediatric Dpt, San Paolo Hospital, ASST Santi Paolo-Carlo, Milan, Italy; 5 Pediatric Primary Care, TA 1, Taranto, Italy; 6 Pediatric Primary Care, ULSS 3, Venice, Italy; 7 Institute for Maternal and Child Health, IRCCS Burlo Garofolo, Via dell’Istria 65/1, 34100 Trieste, Italy

**Keywords:** Breastfeeding, Survey, Breastfeeding support, Hospitalized children

## Abstract

**Background:**

In 2021, the Task Force on Breastfeeding of the Italian Ministry of Health released a document calling for the provision of breastfeeding support in case of re-hospitalization of the child after birth. Since type and quality of breastfeeding support during re-hospitalization in Italian Pediatric Units (PUs) is largely unknown, the Breastfeeding Section of the Italian Society of Pediatrics (TASIP) conducted an ad hoc national survey.

**Methods:**

In March 2023, a specifically designed electronic questionnaire was sent to the Directors of 328 PUs, who were requested to fill it online.

**Results:**

Data from 161 PUs were received, with a response rate of 48.7%. Our results highlighted that 18.6% of units do not provide training on breastfeeding for healthcare professionals and 46% of PUs lack of an ad hoc policy on breastfeeding support in case of re-hospitalization of the child. Although 88.2% of PUs provide breast pumps to the mothers of the re-hospitalized young children, 34.8% lack of a protocol on the storage of expressed breast milk.

**Conclusions:**

Breastfeeding support for the mothers of hospitalized breastfed young children appears to be suboptimal in Italian PUs. Interventions aimed to structure and improve the quality of breastfeeding support for the mother-child dyad are needed, particularly developing protocols and providing a training on breastfeeding to the majority of healthcare professionals.

**Supplementary Information:**

The online version contains supplementary material available at 10.1186/s13052-023-01573-x.

## Background

Breastfeeding represents the ideal source of nutrition for most newborns and infants, and a beneficial component of lifestyle for mothers. Not breastfeeding, either due to early suspension or failure to start, can increase the risk of developing infectious diseases, and Sudden Infant Death Syndrome (SIDS) (and leukemia, type 2 diabetes, overweight/obesity later in life) in children, and the risk of breast and ovarian cancers in mothers [1]. The World Health Organization [2], the Italian Ministry of Health (MoH) [3] and the Pediatric Scientific Societies [4, 5] recommend to exclusively breastfeed for the first 6 months of life, and to continue breastfeeding, together with complementary foods, up to the second year of life and beyond, according to the desire of the mother and the child.

The separation of the mother-child dyad, including the case of re-hospitalization, is associated with significant stress for both the nursing mother and her child [6], as demonstrated by the alteration of the physiological circadian secretion of cortisol [7], with a negative impact on the physiology of lactation, possibly leading to discontinuation of breastfeeding [8].

Given the immuno-biological properties of breast milk, keeping mother and child together during re-hospitalization would maintain breastfeeding, thus providing protection against nosocomial infections, particularly of viral etiology [9, 10]. Whenever direct breastfeeding is not possible, milk production might be maintained by either mechanical or manual breast milk expression [11, 12]. The Academy of Breastfeeding Medicine [13] and, in Italy, the MoH provide organizational and procedural indications to support the continuation of breastfeeding in case of re-hospitalization of the mother and/or of the child [14].

The present study, conducted by the Breastfeeding Section of the Italian Society of Pediatrics (TASIP), explores current practices related to the support of breastfeeding in Italian Pediatric Units (PUs), following MoH’s indications.

## Methods

In March 2022, an email invitation was sent to the Heads of all the Italian PUs, asking to participate in a national online Survey on “Breastfeeding and Re-hospitalization”, launched by the TASIP and aimed to collect information on breastfeeding support in case of hospitalization of the newborn/infant, occurring after the hospital discharge at childbirth. An electronic 15 questions’ structured Survey, that required just a few minutes to be filled, was delivered through the platform Google Forms, collecting data on: a) the fasting times used in the PU for breastfed infants/children before an anesthetic and/or a surgical procedure; b) the availability of a protocol on the expression and storage of mother’ own milk; c) the availability of electric and/or manual breast pumps; d) the existence of a dedicated place for breast milk pumping (either inside the PU or in the Neonatal Unit); e) the availability of dedicated refrigerators for mothers’ milk storage; f) the availability of a mother’s bed or a nursing chair in the child’s room; g) the staff’s training on breastfeeding (*Supplementary Material*).

## Results

**Fig. 1:**
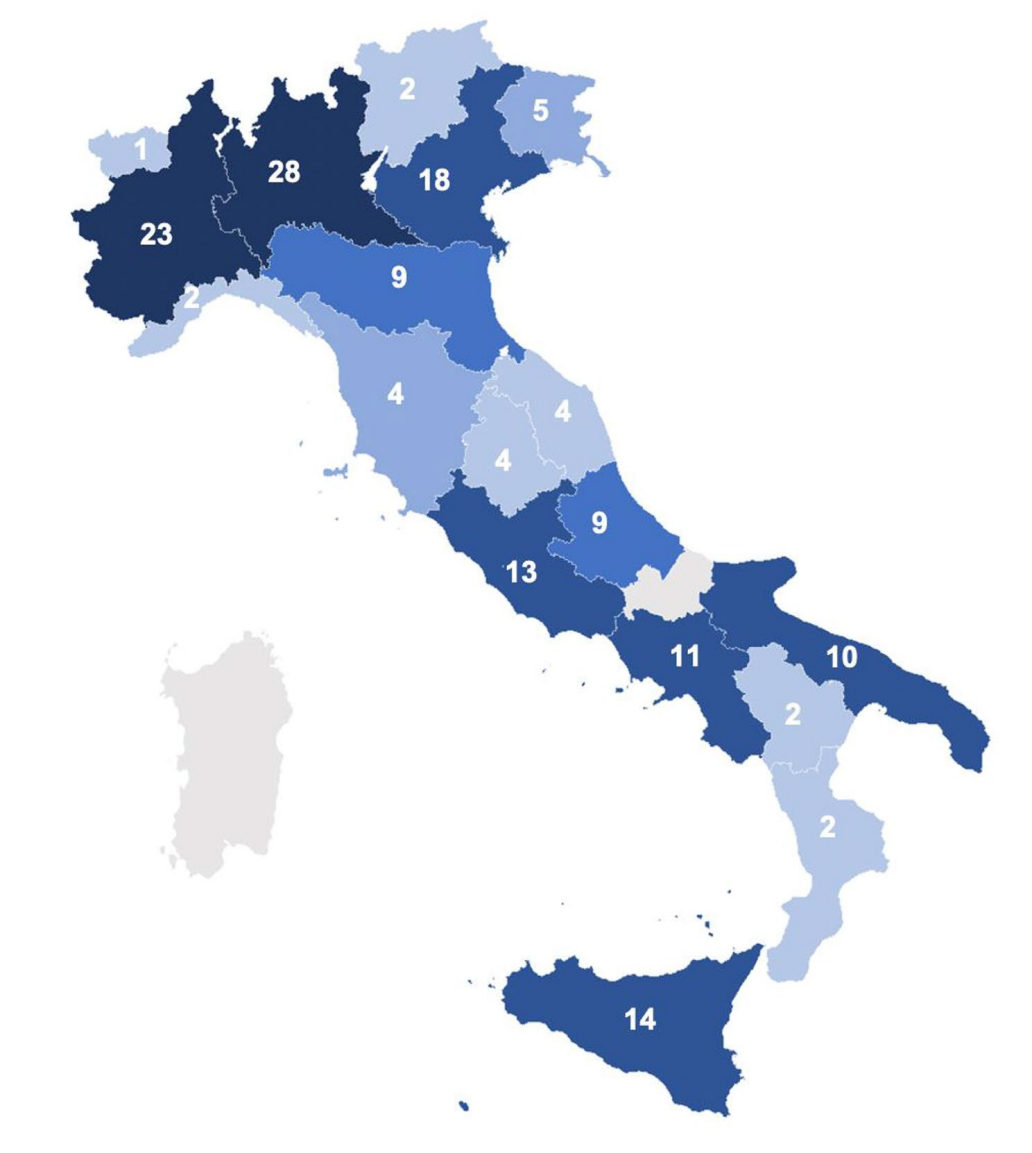
**Geographical distribution of the Pediatric Units that participated to the survey** The picture shows, for each Italian Region, the number of Pediatric Units that answered to the questionnaire on re-hospitalization of breastfed children. No data were available for Molise and Sardinia

The response rate was 48.7% (161 out of 328), with a wide inter-regional variability (range: 22.2–100%), and no response from 2 Regions (Molise and Sardinia). The sample composition was fairly distributed across the national territory (Fig. 1).

Selected characteristic of the PUs that participated to the survey are reported in Table 1. The great majority of PUs have an accommodation capacity of less than 20 beds (86.3%) and admit infants aged less than 6 months (97.5%). Moreover, 34.8% of PUs admit also surgical patients aged < 12 months.

**Table 1:** Characteristics of the Italian Pediatric Units included in the survey

**Accommodation capacity**	**PUs: Percentage (N)**
*< 10 beds*	27.3% (44)
*10–20 beds*	59% (95)
*21–30 beds*	11.2% (18)
*> 30 beds*	2.5% (4)
**Acceptance of infants aged less than 6 months**	**PUs: Percentage (N)**
*Yes*	97.5% (157)
*No*	2.5% (4)
**Acceptance of surgical patients**	**PUs: Percentage(N)**
*Yes*	34.8% (56)
*No*	65.2% (105)

PUs: Pediatric Units

*Organizational support and facilities for zero separation between mother and baby, and for breast feeding maintenance (*Table 2*).*

**Table 2:** Breastfeeding Support for hospitalized sick children in 161 Italian PUs

**Availability of ad hoc protocol on the maintenance of breastfeeding and the use of mother’s milk, in case of hospitalization of a child younger than 2 years**	**N**	**Percentage**
*-Yes*	87	54.1%
*-No*	44	27.3%
*- No, we do not need it*	10	6.2%
*- No, but we plan to develop one*	20	12.4%
**Availability of an ad hoc protocol on the maintenance of breastfeeding and the use of mother’s milk endorsed by Hospital Director**	**N**	**Percentage**
*- Yes*	48/92	52.2%
*- No*	44/92	47.8%
**Availability of breast milk pumps in the PU**	**N**	**Percentage**
*- Yes*	142	88.2%
*-No*	8	5%
*- No, we invite mothers to carry their own from home*	4	2.5%
*- No, we invite mothers to go to the Neonatal Unit to pump off*	7	4.3%
**Type of pumps available in the PU**	**N**	**Percentage**
*- Electric pumps* *- Manual pumps*	123/143 45/122	86.0% 36.9%
**Bottles for collecting and administrating expressed breast milk**	**N**	**Percentage**
*- Are provided by the hospital*	151	93.8%
*- Mothers carry bottles from home*	10	6.2%
**Place where mothers pump off their breast-milk**	**N**	**Percentage**
*- In a dedicated area of the PU*	38	23.6%
*- Besides child’s bed*	112	69.6%
*- In the Neonatal Unit*	11	6.8%
**Availability of a protocol on the storage of expressed breast milk**	**N**	**Percentage**
*- Yes*	105	65.2%
*- No*	56	34.8%
**Storage of the expressed mother’s milk**	**N**	**Percentage**
*- In the shared fridge of the PU*	53	32.9%
*- In a dedicated fridge of the PU*	106	65.8%
*- In a cooler managed by the mothers*	2	1.3%
**Attendance of the PU staff to a credited training course on breastfeeding**	**N**	**Percentage**
*- Yes, only nurses* *- Yes, only physicians*	15 0	9.3% 0.0%
*- Yes, both nurses and physicians*	116	72.0%
*- No*	30	18.7%
**Reasons for not attending a training course on breastfeeding** (more than one answer possible)	**N**	**Percentage**
*- Limited interest/other priorities*	14/31	45.1%
*- Lack of hospital/PU policy*	12/31	38.8%
*- Lack of time*	7/31	22.6%
*- Concurrent organizational problems related to the COVID-19 pandemic*	2/31	6.4%

PU = Pediatric Unit

**Fig. 2:**
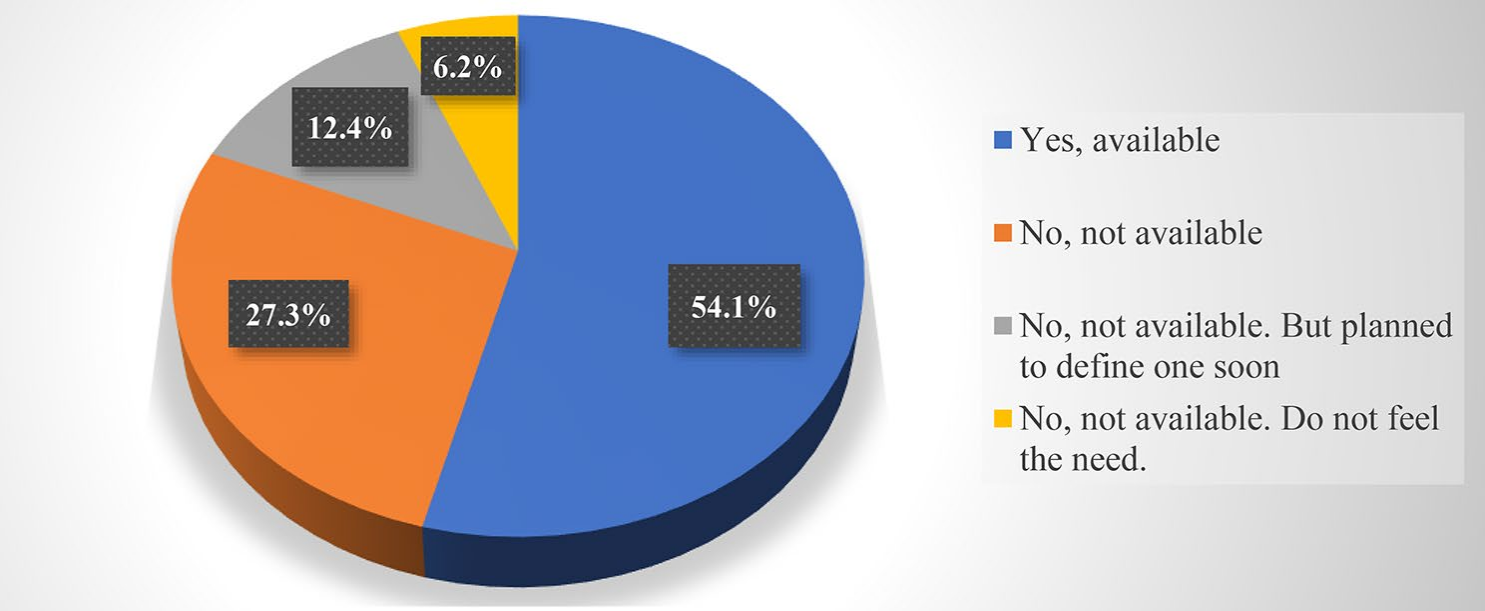
Responses related to the availability of a Pediatric Unit’s protocol on breastfeeding support

- Almost all PUs (92.5%) provide bed or a nursing chair to the mother, in order to room-in with her/his baby, allowing to take care for the baby, rest, breastfeed and, in case, pump own breast milk, with no significant differences between the PUs according to accommodation capacity.
- Only 54.1% of the PUs has an ad hoc protocol regarding breastfeeding and the use of expressed milk in case of re-hospitalization of children aged < 2 years. The accommodation capacity of the PU has no significant impact on the availability of a protocol: a protocol is available in 45.5% of PUs with < 10 beds, in 58.9% of PUs with 10–20 beds, in 44.4% of PUs with 21–30 beds, and in 75% of those with > 30 beds. Almost half of the PUs (45.9%) do not have any protocol on breastfeeding, although 12.4% plan to define one in the short term (Fig. 2). When available, the PU protocol on breastfeeding has been endorsed and supported by the Hospital Director in only 52.2% of cases.
- A specific protocol on the expression and storage of breast milk is lacking in 34.8% of the PUs, with no significant difference according to accommodation capacity (< 10 bed 40.1%; 10–20 beds 32.6%; 21–30 beds 33.3%; >30 beds 0%). Nevertheless, in the great majority of the PUs (88.2%), breast pumps are made available for the nursing mothers (86% electric and/or 36.9% manual). As for the remaining cases, given the absence of breast pumps, the mother is either asked to bring her breast pump from home or offered to use the breast pumps available in the Neonatology Unit (4.3%). The bottles for milk collection are provided by the PUs in 93.8% of cases.
- The milk expression usually takes place inside the PU (in the room where the child is cared for: 69.6%) or in another dedicated area (23.6%), with no significant difference according to accommodation capacity of the PU.
- Concerning storage of expressed breast milk in the PUs, a dedicated refrigerator is available in 65.8%, a non-dedicated refrigerator in 32.9%, while in 1.2% of cases breast milk is stored in a cooler bag directly managed by the mother. No significant differences are detected according to accommodation capacity of the PU.

*Staff training on breastfeeding (*Table 2*).*

- In 72% of the PUs both medical and nursing staff received some training on breastfeeding, while in 9.3% the training involved only the nursing staff. Conversely, in 18.6% of PUs healthcare professionals did not receive any training. The accommodation capacity of the PU has no significant impact on staff training, even if the rate of healthcare professionals that did not receive any training on breastfeeding is lower in PUs with < 10 beds (6.8%) compared to those with > 30 beds and 10–20 beds (25%), or 21–30 beds (11.1%). However, this difference is not statistically significant.
- The reasons reported for not attending breastfeeding courses were (multiple answers were possible): other priorities/limited interest (45.1%), lack of a hospital/PU policy (38.8%), lack of time (22.6%) and, lastly, concurrent organizational problems related to COVID-19 pandemic (6.4%).
- *Fasting time for anesthetic and surgical procedures (*Table 3*).*

**Table 3:** Fasting time for infants undergoing surgical and anesthetic procedures, in Italian PUs

**Fasting time for infants undergoing surgery**	**PUs: Percentage (N)**
*0–2 h*	8.6% (5)
*3–4 h*	48.3% (28)
*5–6 h*	34.5% (20)
*7–8 h*	3.4% (2)
*> 8 h*	5.2% (3)
**Fasting time for infants undergoing anesthetic procedures**	**PUs: Percentage** (**N)**
*0–2 h*	13.3% (8)
*3–4 h*	55.0% (33)
*5–6 h*	25.0% (15)
*7–8 h*	1.7% (1)
*> 8 h*	5.0% (3)

PUs: Pediatric Units

- Fasting time for infants undergoing surgery or anesthetic procedures varied widely among the different PUs. Answers collected in the different PUs are summarized in Table 3.

## Discussion

Maternal decision to breastfeed is influenced by multiple factors, such as personal, demographic, familiar, and socio-economic variables [15–18].

However, factors related to the organization of health facilities should not be underestimated, as confirmed by the drop in breastfeeding rates at discharge from Maternity Hospitals following the sudden change of perinatal practices during the recent COVID-19 pandemic [19, 20]. Particularly, pediatricians play a major role in the organization of healthcare facilities regarding the implementation of policies promoting and supporting lactation and the use of human milk, not only at childbirth but also in case of subsequent hospitalization of the breastfed child [5, 21, 22].

Hospitalization of the breastfed child can interfere with the exclusivity of breastfeeding or even induce a switch to exclusive formula feeding [23]. It has been demonstrated that 51% of mothers of infants hospitalized for bronchiolitis changed their feeding pattern: 20.4% stopped breastfeeding and 14% switched to mixed feedings (breastfeeding plus formula feeding), while 16% noticed a reduction in milk production [24]. According to the mothers’ opinion, the change in feedings’ mode was mainly influenced by lack of support and appropriate advice (63%), severity of the child’s respiratory disease (32%), logistical hospital difficulties (30%), and personal organizational issues (9.3%) [24].

In a recent paper Hookway and Colleagues have explored the experiences of mothers breastfeeding their children in a pediatric hospital setting; challenges to breastfeed a sick child have been documented to be multiple and diverse in nature [25]. Understandably, mothers report the negative impact on breastfeeding not only of the anxiety and depression related to illness severity, but also of suboptimal ward culture on breastfeeding, inadequate staff attitude and lack of psychological and technical support to deal with a complex breastfeeding [25].

Our study shows that 45.9% of Italian PUs lack of a specific protocol addressing maintenance of breastfeeding in the mother-child dyad during re-hospitalization. In particular, in 34.8% of the PUs no specific protocol on the storage of expressed breast milk is available. These shortcomings may be due to a limited cultural investment in supporting breastfeeding, judged not worthy of prioritization or possibly even not essential. Nevertheless, some mothers’ practical needs are met in most cases, since they are provided with a breast pump in 82.8% of the PUs, and with a bed or a long chair that enable to express breast milk at child bedside. However, we were surprised to find out that in 8% of PUs a bed/chair for the mother is not available. Although the reasons underlying this data have not been investigated, it is possible that this finding is linked to the parents’ inability to access the ward. Regardless of the reasons, we believe that the choice to separate the caregiver from a pediatric patient during hospitalization is never justified and should always be considered as inappropriate.

It is noteworthy that 18.6% of the PUs provide no specific training of healthcare professionals on breastfeeding, despite data showing that educated health professionals are more effective in promoting, protecting and supporting breastfeeding [26–29].

Finally, even in hospitals where pediatric anesthesia and/or surgery are performed, a consistent indication on fasting times is lacking, despite the availability of dedicated guidelines [30]. We have found that in 30% of the PUs a fasting of more than 4 h before anesthesia is required, even if 2 h are considered sufficient in breastfed infants. Moreover, in 8.6% of the PUs fasting before surgery is longer than 6 h, despite 4 h are considered sufficient even for formula fed infants. Prolonged fasting is not only unnecessary, particularly in breastfed infants, but it is also inadvisable due to the increased risk of ketosis and hypotension [31].

To the best of our knowledge, this is the first survey evaluating breastfeeding support practices in a large and geographically representative sample of Italian PUs.

However, the study also has also some limitations. Firstly, the response rate seems relatively low, being a 48.7%. Nevertheless, given the design of a survey-based study, a response rate between 40% and 50% is acceptable, to reduce the likelihood of non-responder bias. [32–34]. Therefore, we are confident that a nearly 50% response rate with a homogeneous distribution on the national territory, represents a reliable sample to provide a representative overview of the Italian situation.

Secondly, considering that participation in the present study was voluntary, it is possible that those who answered were the most motivated in supporting breastfeeding. Consequently, our results might overestimate among responders the commitment on the promotion and the support of breastfeeding in Italian PUs, thus giving a more optimistic picture than the reality.

In addition, the questionnaire did not include any question allowing to discriminate between Pediatric/Neonatology Units and Pediatric Units. For this reason, we are unable to define whether the presence of a Neonatology Unit could have influenced our results, particularly in terms of a stronger valorization of breastfeeding and mother’s milk.

## Conclusions

Although most Italian PUs show some attention in supporting breastfeeding in case of a re-hospitalization of the child, we have identified areas for possible improvement. The development of a uniform breastfeeding policy is particularly needed, since it might foster a specific training of healthcare professionals, and the implementation of hospital practices and protocols to maintain breastfeeding while treating the hospitalized sick child.

Ultimately, promoting breastfeeding of the hospitalized child would clearly show that the PUs factually recognize the value of breastfeeding and the use of breast milk, beyond any manneristic position statement.

### Electronic supplementary material

Below is the link to the electronic supplementary material.

Supplementary Material 1

## Data Availability

The datasets used and/or analyzed during the current study are available from the corresponding author on reasonable request.

## Abbreviations

MoH: Ministry of Health
PUs: Pediatric Units
SIDS: Sudden Infant Death Syndrome
TASIP: Breastfeeding Section of the Italian Society of Pediatrics
WHO: World Health Organization
