# Supplementary material for: Breastfeeding support among re-hospitalized young children: a survey from Italy
Source: Ital J Pediatr. 2024 Jan 8;50:3. doi: 10.1186/s13052-023-01573-x (PMC10775476; doi:10.1186/s13052-023-01573-x)
Supplement: Supplementary file 1 — Supplementary Material 1 [file 13052_2023_1573_MOESM1_ESM.docx]

**Survey of the Breastfeeding Technical Table of the Italian Society of Pediatrics**

**Breastfeeding support in case of re-hospitalization of the breastfed infant**

**Questionnaire for the Directors of the Italian Pediatric Units**

**1. In which region is your Pediatric Unit located?**

**2. In which province is your Pediatric Unit located?**

**3. In your Pediatric Unit, do you also admit children under the age of 6 months?**

1. Yes
2. No

**4. Does your Pediatric Unit have an *ad hoc* protocol on the maintenance of breastfeeding and on the use of mother’s expressed breast milk, in case of hospitalization (ordinary hospitalization) of a child (<2 years) who is breastfed in the event of illness or investigations?**

1. Yes
2. No
3. No, we do not need it
4. No, but we plan to develop one

**5. If the answer to the previous question is "yes", is the protocol endorsed by the Hospital Director?**

1. Yes
2. No

**6. Are breast milk pumps available in your Pediatric Unit?**

1. Yes
2. No
3. No, we invite mothers to carry their own from home
4. No, we invite mothers to go to the Neonatal Unit to pump off

**6a.     If you answered "yes" to question 6, are these electric breast pumps?**

1. Yes
2. No

**6b.     If you answered "yes" to question 6, are these manual breast pumps?**

1. Yes
2. No

**7. Bottles for collecting and administering breast milk:**

1. Are provided by the hospital
2. Mothers carry bottles from home

**8. In your Pediatric Unit, where do mothers pump breast milk?**

1. In a dedicated area of the Pediatric Unit
2. Besides child’s bed
3. In the Neonatal Unit

**9. Does your Pediatric Unit have a protocol on the storage of expressed breast milk?**

1. Yes
2. No

**10. Are surgeries performed in your Pediatric Unit?**

1. Yes
2. No

**10a. If you answered "yes" to question 10, what is the fasting time for an infant before surgery?**

a) 0-2 hours

b) 3-4 hours

c) 5-6 hours

d) 7-8 hours

e) >8 hours

**10b. If you answered "yes" to question 10, what is the fasting time for an infant before an anesthetic procedure?**

a) 0-2 hours

b) 3-4 hours

c) 5-6 hours

d) 7-8 hours

e) >8 hours

**11. The mother's expressed breast milk is kept:**

1. In the shared fridge of the Pediatric Unit
2. In a dedicated fridge of the Pediatric Unit
3. In a cooler managed by the mother

**12.   Has the pediatric staff of your Pediatric Unit attended a training course dedicated to breastfeeding (in presence or online)?**

1. Yes, only nurses
2. Yes, only physicians
3. Yes, both nurses and physicians
4. No

**13. If the answer to question 12 is "no", please indicate why (more than one answer is possible):**

1. Lack of time
2. Limited interest
3. Other priorities
4. Lack of hospital/Pediatric Unit policy
5. Not included in the Department agenda
6. Other

**14. Does the nursing mother have in the child’s room a bed or an armchair to rest and breastfeed?**

a) Yes

b) No

**15. Please indicate the number of hospital beds in your Pediatric Unit:**

a) <10

b) 10-20

c)21-30

d) >30
